# Whole-genome sequencing of endangered Zhoushan cattle suggests its origin and the association of MC1R with black coat colour

**DOI:** 10.1038/s41598-021-96896-2

**Published:** 2021-08-30

**Authors:** Lihua Jiang, Tetsuo Kon, Chunyan Chen, Ryota Ichikawa, Qiyuan Zheng, Liyi Pei, Ikuyo Takemura, Lauden Hagai Nsobi, Hiromasa Tabata, Hao Pan, Yoshihiro Omori, Atsushi Ogura

**Affiliations:** 1 National Engineering Research Center of Marine Facilities Aquaculture, Zhejiang Ocean University, Zhoushan, 316022 China; 2 Genomic Diversity Laboratory, Graduate School of Bioscience, Nagahama Institute of Bioscience and Technology, Nagahama, Shiga 526-0829 Japan; 3 Laboratory of Functional Genomics, Graduate School of Bioscience, Nagahama Institute of Bioscience and Technology, Nagahama, Shiga 526-0829 Japan; 4 Zhoushan Sanxing Zhoushan Cattle Protection Institute, Zhoushan City, 316000 China

**Keywords:** Evolutionary biology, Phylogeny, Animal breeding, Evolutionary genetics, Phylogenetics, Population genetics, Conservation biology

## Abstract

Zhoushan cattle are an endangered cattle breed in the Zhoushan islands in China. Since Zhoushan cattle have been bred in isolation, they show unique characteristics, such as dark black coat colour. However, no studies have been conducted on the genome of Zhoushan cattle. Here, we performed whole-genome sequencing of seven individuals of Zhoushan cattle and nine cattle in Wenling, geographically close to the Zhoushan islands. By integrating our data and publicly-available data, we found that Zhoushan cattle are genetically highly similar to *Bos indicus* cattle in south-eastern China. Furthermore, by identifying the genomic regions shared between Zhoushan cattle and Angus cattle, a *Bos taurus* breed, we found that the p.F195L mutation in melanocyte-stimulating hormone receptor (MC1R) could be associated with their dark black coat colour. Taken together, our results provide a valuable resource for characterising the uniqueness of Zhoushan cattle.

## Introduction

Chinese cattle are distributed throughout the country and are among the most critical components of world cattle due to their large number and abundant breed types^1–3^. Chinese cattle have long been used as draught animals, enhancing their pronounced merits in parasite resistance, utilisation of roughage-based diets, and tolerance to environmental challenges. Since cattle with a yellow coat colour are the overall majority in China, Chinese cattle have long been collectively called yellow cattle^4^. Likewise, black cattle are the minority.

Zhoushan cattle are a unique Chinese cattle breed that has been bred in the Zhoushan islands^5^. The Zhoushan islands are in the East China Sea, which is physically isolated away from the Chinese mainland by the sea. The Chinese mainland areas near the Zhoushan islands include Wenling and Shanghai. Zhoushan cattle have been bred on the Zhoushan islands for at least 300 years^1,5^. Zhoushan cattle show a dark black coat colour, curving and twisted horns, and large body size^2,5^. Zhoushan cattle have been widely used to cultivate paddy fields, but recently they have been replaced by mechanical cultivation and are in danger of extinction^5^. The population size was estimated to be approximately 5000 in 1980, and now the number has decreased further^2^.

Modern domesticated cattle consist of two major lineages, *Bos indicus* and *Bos taurus*^6^. These cattle are derived from independent domestications of the same progenitor species, *Bos primigenius*^6^. There are distinct differences in physical characteristics between these two lineages. *B. taurus* is a breed of cattle that originated from cattle in Europe^6^. The body of *B. taurus* is smooth with no protrusions and has tightened skin. Some typical breeds of *B. taurus,* such as Black Angus and Kobe cattle have a dark black coat. Although some non-black Angus breeds are known, all Angus breeds are derived from the cattle breed with black coat colour. Conversely, *B. indicus* is a breed of cattle that originated from cattle in South Asia^3,7^. *B. indicus* shows distinctive physical characteristics, such as a hump on the back, excess skin across the entire ventral midline, especially around the neck (throatlatch), chest (dewlap), and navel, larger ears, and shorter coat hair to cope with warmer climates than Europe^8^. These traits are not prominent in *B. taurus*. It was suggested that *B. taurus* was brought into East Asia from West Asia during the late Neolithic period, and *B. indicus* later was imported from India to East Asia^9,10^. Recent genome-wide analysis of Chinese cattle revealed that *B. taurus* is mainly located in east China and that *B. indicus* is primarily located in south China^3^. In north-central China, there are various hybrids between *B. taurus* and *B. indicus*^3^.

The origin of Zhoushan cattle is poorly understood because there are few records of their origin. Since Zhoushan cattle has not been genetically studied, the mainland cattle genetically closest to Zhoushan cattle are still unknown. In particular, it is fascinating to investigate whether the breed that is genetically closest to Zhoushan cattle is *B. taurus* or *B. indicus* or a hybrid thereof. Furthermore, Zhoushan cattle look like both *B. taurus* and *B. indicus*; considering that Zhoushan cattle have a hump on the back, some people propose that Zhoushan cattle are *B. indicus*^5^. Others suggest that Zhoushan cattle are *B. taurus* because the dewlap is less prominent, they show the dark black coat colour similar to Kobe cattle in Japan, and the Zhoushan islands are geographically close to Japan across the sea. If we identify the breed that is genetically closest to Zhoushan cattle, we could infer their origin. However, thus far, there has been no research on Zhoushan cattle's genome. Here, we performed whole-genome sequencing of seven individuals of Zhoushan cattle and nine Wenling cattle individuals, which are a local breed in Wenling and show typical characteristics of *B. indicus*. Wenling is a region of the Chinese mainland located south of Zhoushan islands and close to Zhoushan islands. Combined with the publicly-available genomic data of other cattle breeds, we performed population analyses with Zhoushan cattle and other domesticated cattle.

## Results

### Whole-genome sequencing of Zhoushan cattle and Wenling cattle populations

**Figure 1:**
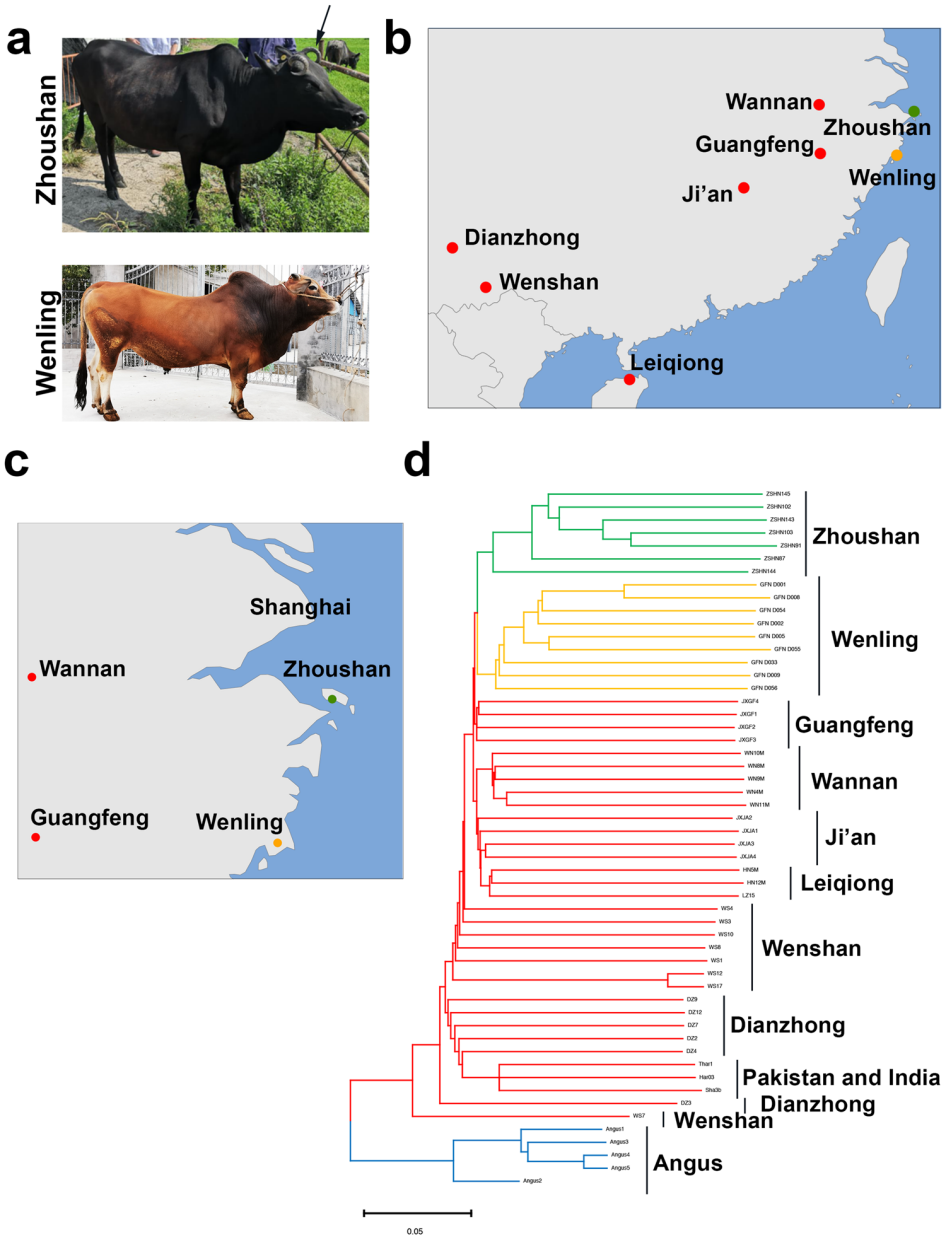
Phylogenetic analysis of Zhoushan cattle and other cattle breeds. (**a**) Gross appearance of Zhoushan (upper panel) and Wenling cattle (lower panel). Note that Zhoushan cattle have a dark black coat colour. The arrow indicates the curving horn of Zhoushan cattle. (**b**) Geographic map indicating the origins of Zhoushan (green dot) and Wenling (orange dot) cattle analysed in this study. We also examined other Chinese cattle (red dots) whose genome sequencing data were available. (**c**) Regional map around the Zhoushan islands. Wenling, Wannan, and Guangfeng are mainland regions close to the Zhoushan islands. (**d**) Neighbour-joining tree of the 54 domesticated cattle. The scale bar represents pairwise distances between different individuals. The maps were constructed by R^38^ and R packages of maps v3.3.0 (https://cran.r-project.org/web/packages/maps) and mapdata v2.3.0 (https://cran.r-project.org/web/packages/mapdata).

We collected seven individuals of Zhoushan cattle (Fig. 1a, upper panel). We also collected nine individuals of Wenling cattle (Fig. 1a, lower panel). Wenling cattle have a prominent hump on the back, dewlap, and larger ears, suggesting that its genetic background is largely *B. indicus* (Fig. 1a, lower panel). We performed whole-genome sequencing of these samples. To resolve their phylogenetic positions and interrelationships within domesticated cattle, we combined our data of 16 cattle individuals with publicly-available whole-genome sequencing data of five individuals from the Angus breed, a typical *B. taurus* in Europe, and 33 individuals from nine breeds with genetic backgrounds similar to *B. indicus*^3^, giving a total of 54 individuals (Fig. 1b, c; Table S1). We performed read trimming and aligned the trimmed reads to the UOA_Brahman_1 assembly of the cattle genome^11^. This assembly represents the maternal haplotype of an F1 hybrid of Brahman cattle (dam) and Angus (sire)^11^. After variant calling and filtering, we identified 32,970,327 single-nucleotide polymorphisms (SNPs) and 3,331,322 small indels. Based on this genomic variant information, we conducted the population genomic analyses.

### Genetic relationship between Zhoushan cattle and other domesticated cattle

To reveal the phylogenetic positions and interrelationships of Zhoushan and other domesticated cattle, we performed population genomic analyses on 54 cattle individuals. First, we calculated the pairwise evolutionary distance between individuals and generated a neighbour-joining (NJ) tree to reconstruct the phylogenetic relationships between individuals of Zhoushan and other domesticated cattle (Fig. 1d). In the NJ tree, cattle clustered consistently with their geographical location (Fig. 1d). Angus individuals formed a sister group to all other individuals, including Zhoushan cattle, Wenling cattle, and other *B. indicus* (Fig. 1d). The individuals of Zhoushan and Wenling cattle formed monophyletic groups and were sisters to each other (Fig. 1d). The cattle in Guangfeng formed another monophyletic group and were sisters to both Zhoushan and Wenling cattle (Fig. 1d). Cattle in Wannan, Ji'an, and Leiqiong formed a single group, sister to the cattle of Zhoushan, Wenling, and Guangfeng (Fig. 1d). Zhoushan, Wenling, Guangfeng, Wannan, and Ji'an are geographically close to each other (Fig. 1b, c). The cattle of Dianzhong and Wenshan, which are in the south part of China, were distant from them (Fig. 1d). Cattle in Pakistan and India were located near the root of the phylogenetic tree (Fig. 1d). The branch lengths of Zhoushan cattle were shorter than other *B. indicus* cattle, suggesting the reduced genetic diversity of Zhoushan cattle (Fig. 1d).

**Figure 2:**
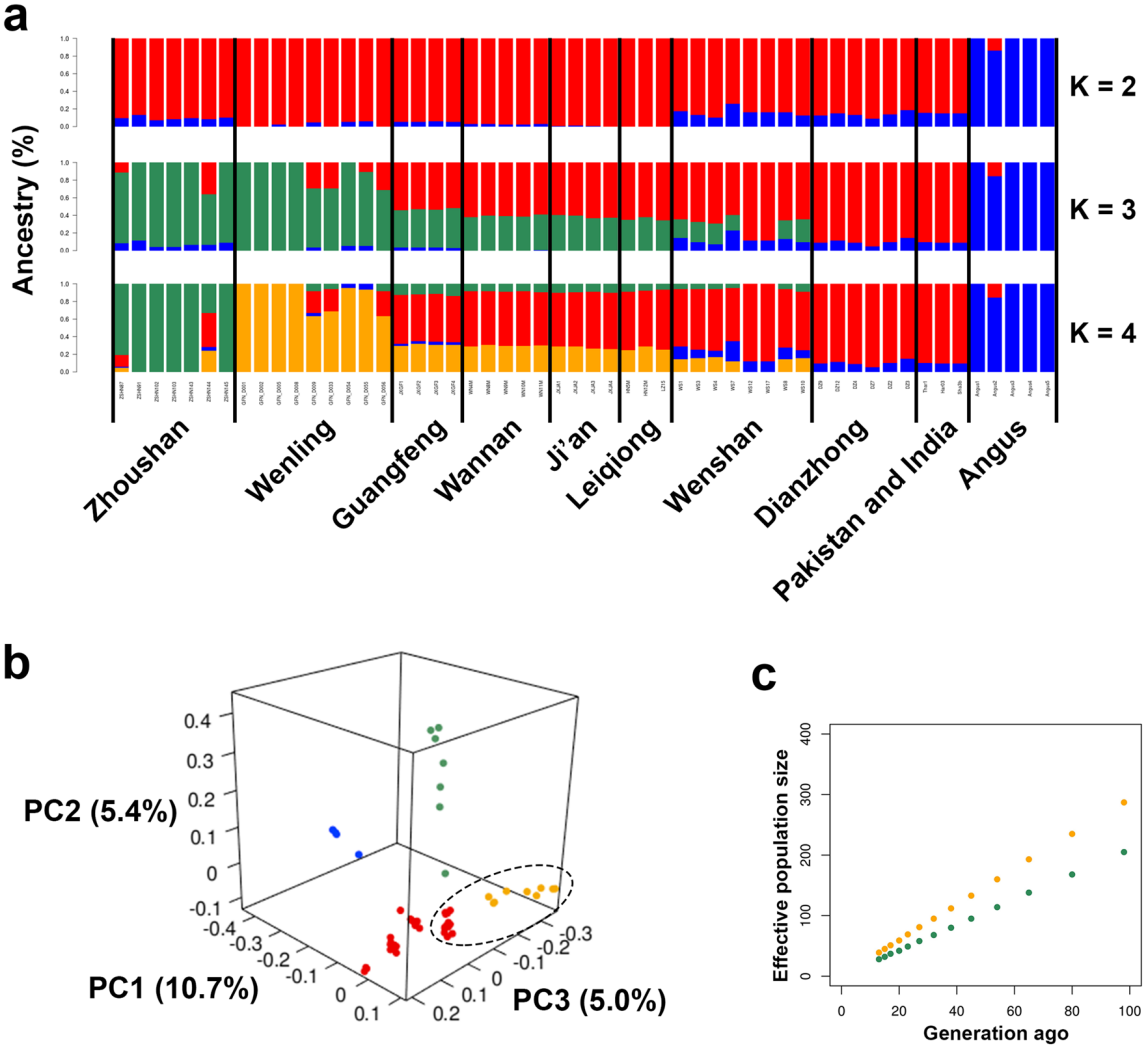
Admixture and principal component analysis of Zhoushan cattle and other cattle breeds. (**a**) Admixture plot (K = 2, 3, 4) for the 54 cattle individuals. Each individual is shown as a vertical bar divided into K colours. (**b**) PCA plot showing the genetic structure of the 54 cattle individuals. The degree of explained variance is given in parentheses. Colours reflect the geographic regions of sampling in Fig. 1d. The cluster composed of cattle in Wenling, Guangfeng, Wannan, Ji'an, and Leiqiong is highlighted in the black dotted ellipse. (**c**) Estimate of the effective population sizes of Zhoushan (green) and Wenling (orange) cattle over the past 100 generations.

To estimate the relatedness between Zhoushan and other domesticated cattle, we performed unsupervised clustering analysis with ADMIXTURE v1.3.0 software (https://dalexander.github.io/admixture/index.html)^12^. At K = 2, Angus cattle were distinct from all other cattle (Fig. 2a). At K = 3, Zhoushan and Wenling cattle were newly segregated from other cattle, suggesting that these two cattle breeds are genetically close to each other (Fig. 2a). The cattle of Guangfeng, Wannan, Ji'an, Leiqiong, and Wenshan had intermediate genetic structures between Zhoushan cattle and Dianzhong cattle (Fig. 2a). At K = 4, Zhoushan cattle and Wenling cattle were separated from each other (Fig. 2a).

To infer the population structure of cattle individuals analysed in this study, we conducted principal component analysis (PCA). The top three principal components accounted for 21.1% of the total variance (Fig. 2b). In the first component of PCA, Angus individuals were separated from all other cattle (Fig. 2b). Additionally, cattle of Wenling, Guangfeng, Wannan, Ji'an, and Leiqiong formed a cluster (dotted ellipse in Fig. 2b). In the second component of PCA, individuals of Zhoushan cattle were separated from all other cattle (Fig. 2b). In the third principal component, Wenling cattle individuals were separated from all other cattle (Fig. 2b).

We estimated the trends of the effective population size of Zhoushan and Wenling cattle over the past 100 generations (Fig. 2c). Both populations showed decreasing trends of effective population sizes (Fig. 2c). The effective population size of Zhoushan cattle was estimated to be smaller than that of Wenling cattle, suggesting the effect of island isolation on the genetic diversity of Zhoushan cattle (Fig. 2c).

### Detection of candidate genes associated with dark black coat colour of Zhoushan cattle

**Figure 3:**
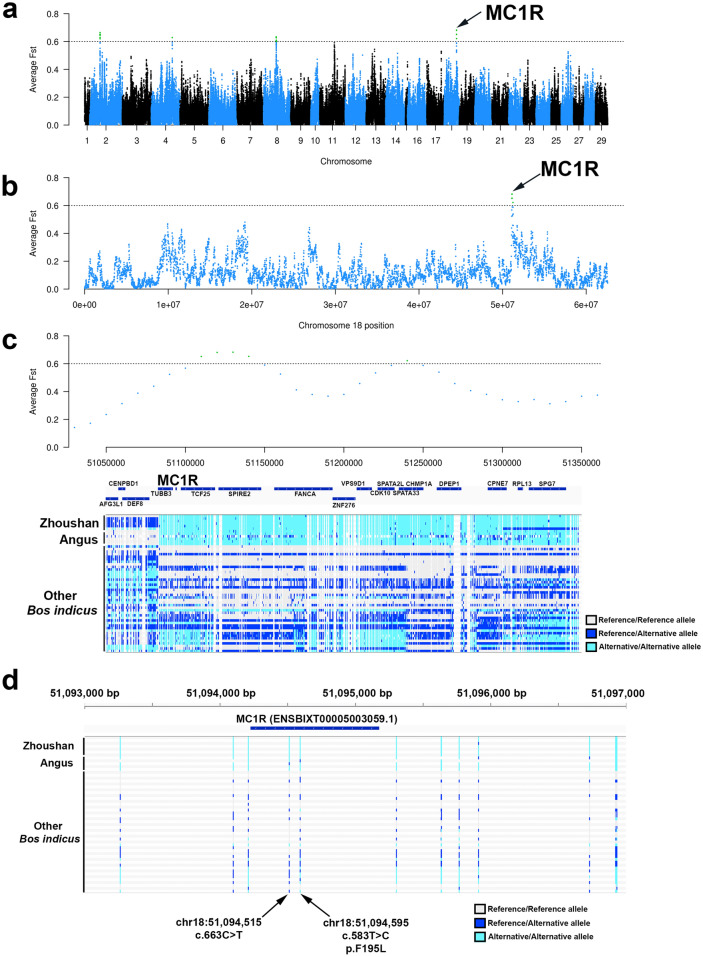
Genomic regions associated with dark black coat colour of Zhoushan cattle. (**a**) Manhattan plot for average Fst values in 40 kb windows with 10 kb steps between Zhoushan cattle plus Angus and other *B. indicus*. A region with an average Fst of more than 0.6 is coloured in green. The arrow indicates the highest peak. The x-axis represents chromosomal positions, and the y-axis represents the average Fst values. (**b**) Manhattan plot on chromosome 18 for average Fst values in 40 kb windows with 10 kb steps between Zhoushan cattle, Angus, and other *B. indicus*. (**c**) Regional plot around the MC1R gene. The genotype of each individual at each variant site is shown. The genotype homozygous for the reference allele is coloured grey. Heterozygous variants are coloured blue. The homozygous genotype for alternative alleles is coloured light blue. Note that homozygous genotypes for alternative alleles are enriched in Zhoushan and Angus cattle in this region. (**d**) Regional plot showing the mutations around MC1R gene.

**Figure 4:**
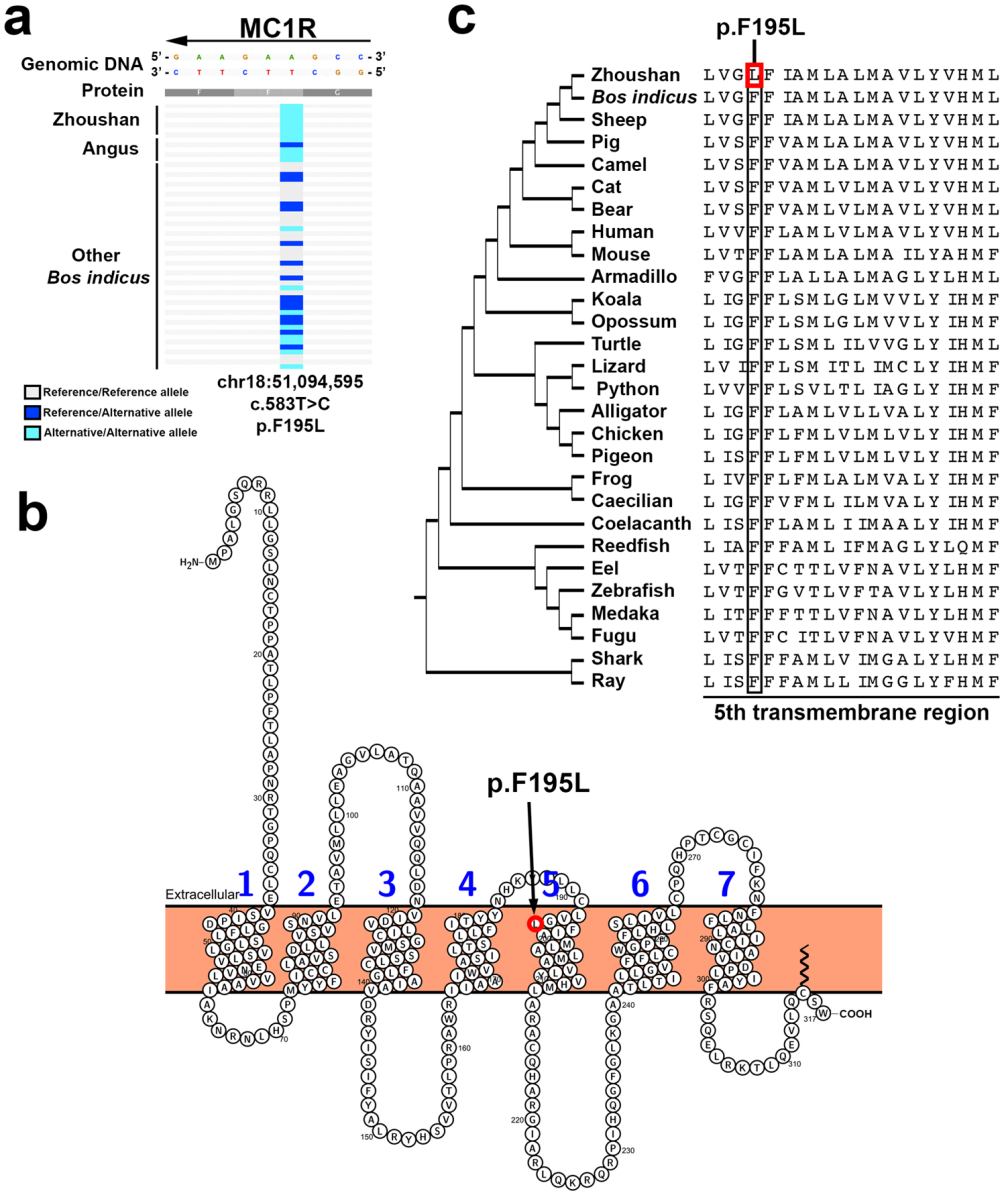
Secondary structure of MC1R and protein sequence alignment of MC1R orthologs. (**a**) Regional highlight of the c.583 T > C mutation of MC1R. The genomic region from 51,094,590 to 51,094,598 bp on chromosome 18 is shown. Note that MC1R is located on the reverse strand. (**b**) Secondary structure of MC1R. MC1R is a seven-transmembrane receptor. The p.F195L mutation is located in the 5th transmembrane region and enclosed by the red circle. This figure is generated by using the Protter server application^39^. (**c**) Multiple sequence alignment of MC1R orthologs. The black rectangle highlights the 195th phenylalanine residues. The red rectangle encloses the p.F195L mutation in Zhoushan cattle. The cladogram of the species is shown to the left of the species name. The cladogram topology is derived from a previous study^40^.

To identify putative genes associated with the dark black coat colour of Zhoushan cattle, we searched genomic regions where the same mutations were shared between Zhoushan cattle and Angus cattle. To achieve this, we calculated the average fixation index (Fst) values in 40 kb windows with 10 kb steps (Fig. 3a). We identified four peaks of Fst at chromosomes 2, 4, 8, and 18 (Fig. 3a). Among these peaks, the highest peak of Fst was identified in the region from 51.05 to 51.35 Mbp on chromosome 18 (Fig. 3a, b). This region contains 18 genes (Fig. 3c). We searched for genes that have mutations altering the amino acid sequence and have been reported to be involved in the regulation of coat colour. Among these 18 genes, only the gene of melanocyte-stimulating hormone receptor (MC1R) is known to involved in the regulation of coat colour^13–15^. Therefore, we regarded MC1R as a strong candidate gene associated with the dark black coat colour of Zhoushan and Angus cattle (Fig. 3c). This gene is located in the region between 51,094,227 bp and 51,095,177 bp on chromosome 18. MC1R is expressed in the skin melanocyte and plays a crucial role in regulating animal coat colour formation^16^. Mutations of MC1R have been reported to be associated with black coat colour in some animals, such as cattle^17^, sheep^16^, pigs^18^, reindeer^19^, and geese^20^. In the protein-coding region of MC1R, we identified one missense mutation (c.583T > C, p.F195L) and one synonymous mutation (c.663C > T) (Figs. 3d, 4a). The missense mutation is located in the fifth transmembrane region of MC1R (Fig. 4b). All seven Zhoushan cattle were homozygous for the missense mutation (Figs. 3d, 4a). Four of five Angus individuals were homozygous for the missense mutation, and the remaining one was heterozygous for the missense mutation (Figs. 3d, 4a). Conversely, only 19% (8/42) and 33% (14/42) of *B. indicus* individuals were homozygous or heterozygous, respectively, for the missense mutation (Figs. 3d, 4a). The remaining 48% (20/42) of individuals of *B. indicus* were homozygous for the wild-type allele (Figs. 3d, 4a). We also found that the p.F195L mutation is also present in MC1R of Black Angus (accession number: ABX83563.1) in the NCBI Protein database (Fig. S1). Furthermore, we identified 15 upstream variants and three downstream variants in the intergenic regions between neighbouring genes (Table S2).

To characterise the missense mutation of MC1R (c.583T > C, p.F195L) found in Zhoushan and Angus cattle, we estimated the degree of evolutionary conservation of the 195th phenylalanine of MC1R. We obtained various MC1R orthologs of vertebrates from eight eutherian mammals, two marsupial mammals, four reptiles, two birds, two amphibians, one lobe-finned fish, one polypterus fish, four teleost fish, and two cartilaginous fish (Table S3). We aligned these 26 sequences with MC1R of Zhoushan cattle and *B. indicus* (Fig. 4c). This analysis revealed that the 195th phenylalanine of MC1R is highly conserved among vertebrates (Fig. 4c).

**Figure 5:**
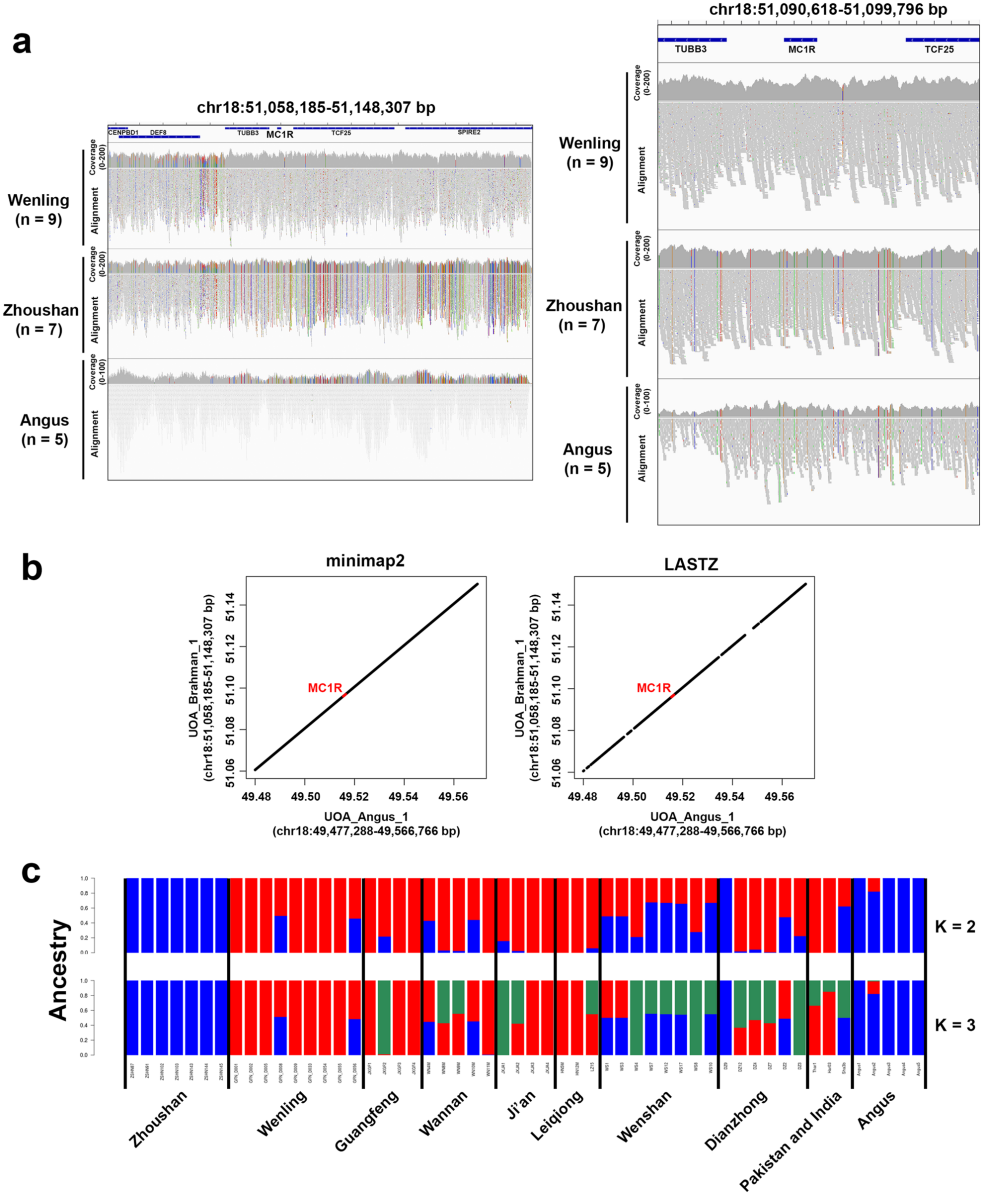
Read depth distribution, genome alignment and admixture analysis of the MC1R region. (**a**) Read depth distributions in the MC1R region. The left panel shows the read depth distributions in the region from 51,058,185 to 51,148,307 bp on chromosome 18. The right panel shows the read depth distributions in the region from 51,090,618 to 51,099,796 bp on chromosome 18. For each breed, the sequencing reads were integrated. The first track represents read depth distribution in each breed, and the second track represents read alignments to the reference genome. For a given base position, if the base call in the sequencing read and the corresponding base in the reference genome are different, adenine is shown in green, thymine in red, guanine in orange, and cytosine in blue. (**b**) Dot plots showing the genome alignments of the MC1R regions of the UOA_Angus_1 assembly (chr18:49,477,288–49,566,766 bp) and the UOA_Brahman_1 assembly (chr18:51,058,185–51,148,307 bp). The left panel shows the genome alignment by minimap2 aligner and the right one shows the genome alignment by LASTZ aligner. The region corresponding to the MC1R gene body is highlighted in red. (**c**) Admixture analysis of the MC1R region. The SNPs located in the MC1R region (chr18:51,058,185–51,148,307 bp) were collected and subjected to admixture analysis. The order of the samples is the same as in Fig. 2a.

Furthermore, we verified whether any larger structural variants are spanning the MC1R region (chr18:51,058,185–51,148,307 bp) of Zhoushan cattle and Angus. If there are large structural variants in this region for these breeds, we should see regions where the read depth distributions are different among the groups. We assessed the integrated read depth distributions of Wenling cattle (n = 9), Zhoushan cattle (n = 7) and Angus (n = 5) (Fig. 5a). The read depth distribution was very similar among the three groups suggesting that there are not large structural variants spanning the MC1R region in these breeds (Fig. 5a). We also collected the sequence reads mapped to this region, and performed BreakDancer to detect structural variants^21^. However, no structural variants were detected in this region in any breeds. Moreover, we compared the reference genome sequence in MC1R region of the UOA_Brahman_1 assembly and that of the UOA_Angus_1 assembly^11^. The UOA_Brahman_1 assembly represents the maternal haplotype of an F1 hybrid of Brahman cattle (dam) and Angus (sire), and the UOA_Angus_1 assembly represents its paternal haplotype^11^. The results showed that the genome sequence in the MC1R region are highly preserved between these two assemblies (Fig. 5b).

Finally, we deduced the origin of the MC1R haplotype in Zhoushan cattle. We collected the SNPs located in the MC1R region (chr18:51,058,185–51,148,307 bp) from all individuals and performed admixture analysis using these SNPs. The result showed that Zhoushan cattle and Angus shared highly similar genetic components (Fig. 5c). However, the other individuals of *B. indicus* showed genetic components that differed from both Zhoushan cattle and Angus (Fig. 5c). These results suggest that the MC1R haplotype in Zhoushan cattle is derived from *B. taurus*, even though the genome of Zhoushan cattle as a whole is that of *B. indicus*.

## Discussion

This study conducted whole-genome sequencing of seven individuals of Zhoushan cattle and nine Wenling cattle individuals. By analysing our data and publicly-available data together, we showed that Zhoushan cattle are genetically similar to Wenling cattle, followed by cattle in Guangfeng, Wannan, and Ji'an (Figs. 1d, 2a, b). Since these cattle's genetic background is *B. indicus*^3^, the genetic background of Zhoushan cattle is also largely *B. indicus*. Wenling, Guangfeng, Wannan, and Ji'an are the south-eastern region of China and geographically close to the Zhoushan islands. Since the cattle that are genetically close to the Zhoushan cattle are located geographically close to it, it is possible that the ancestral population of Zhoushan cattle was introduced from the mainland region near the Zhoushan islands. According to previous studies, the ancestral population of Zhoushan cattle might be Tangjiao cattle in Shanghai^5^. Shanghai is a city located north of Zhoushan islands and is geographically very close to the Zhoushan islands (Fig. 1c). Although Tangjiao cattle died out after the late 1980s, there are pictures of Tangjiao cattle showing its dark black coat colour and similar morphological appearance to Zhoushan cattle^1,5^. Whole-genome sequencing of residual genomic DNA of Tangjiao cattle from remaining hair or other tissues would further elucidate the origin of Zhoushan cattle.

Zhoushan cattle was introduced from these mainland areas across the sea to the Zhoushan islands hundreds of years ago and bred in isolation from the mainland until today^1,5^. Although the genetic background of Zhoushan cattle is similar to that of cattle in nearby mainland regions, including Wenling, Guangfeng, Wannan, Ji'an, and Leiqiong, Zhoushan cattle still show a distinct genetic background from these cattle (Figs. 1d, 2a, b). This is probably the result of island isolation for hundreds of years. We also estimated that the effective population size of Zhoushan cattle is decreasing (Fig. 2c). This is consistent with the previous report that island isolation is associated with reducing genetic diversity in animals^22^. Random genetic drift may cause the unique genetic population structure and the decreasing trend of Zhoushan cattle due to the small population size. Zhoushan cattle can be a valuable study model to analyse the genetic effects of island isolation on mammals' genomic compositions.

The colour of hair, skin, and eyes in animals mainly depends on the quantity, quality, and distribution of two types of melanin granules, eumelanin and pheomelanin, produced by melanocytes^23,24^. Eumelanin is black to brown, and pheomelanin is yellow to reddish^23,24^. We identified MC1R as a candidate gene responsible for the dark coat colour of Zhoushan cattle. MC1R is a G protein-coupled receptor and is expressed in the cell membrane of the skin melanocyte. When MC1R is activated by the melanocyte-stimulating hormone from the pituitary gland, MC1R initiates a downstream signalling cascade that leads to black pigment eumelanin production in the melanocytes^13,14^. We identified the p.F195L mutation in MC1R. So far, there are many reports about mutations of MC1R associated with black coat colour in some animals, such as cattle^17,25^, sheep^16^, pig^18^, reindeer^19^, and geese^20^. For example, the dominant missense mutation of p.L99P results in black coat colour, whereas a frameshift mutation of c.310delG, producing a prematurely terminated MC1R protein (p.Gly104ValfsX53), produces a red coat colour in cattle^17,25^. The mutation of p.F195L is a nonpolar-to-nonpolar conversion and is located in the fifth transmembrane region of MC1R (Fig. 4b). Since this residue is highly evolutionary conserved among vertebrates (Fig. 4c), p.F195L might lead to activation of MC1R. We also identified 15 upstream variants and three downstream variants in the intergenic regions between neighbouring genes. Another possibility is that these intergenic mutations alter the promoter and/or enhancer activity of MC1R, resulting in upregulation of MC1R gene expression. In either case, mutations identified in this study also can be excellent markers for selective breeding for dark black coat colour.

Recently, the mutation of p.F195L was reported as one of six candidate missense mutations associated with the black body colour of cattle^26^. They compared the protein-coding sequences of MC1R in Tharparkar and Karan Fries cattle (Tharparker × Holstein Friesian)^26^. According to the authors, Tharparkar cattle has a predominantly white coat colour, and Karan Fries cattle has a white and black coat colour^26^. They performed molecular cloning of MC1R genes from both cattle and identified six missense mutations, including p.F195L in Karan Fries cattle. Since Holstein Friesian cattle, a *B. taurus* breed, has a white and black coat colour, these mutations can be associated with the generation of the black coat area on Karan Fries cattle^26^. Notably, we only identified p.F195L among these six mutations by the unbiased whole-genome analysis. Therefore, the mutation of p.F195L may be a novel causative mutation of MC1R in Zhoushan and some *B. taurus* cattle with a dark coat colour.

Chinese cattle are one of the most important world cattle compositions because of their large number and abundant breed types. However, a number of local breeds, including Zhoushan cattle, are on the verge of extinction^1,5^. Zhoushan cattle have a unique history of being bred in the Zhoushan islands isolated from the mainland for hundreds of years^5^. Zhoushan cattle are closely related to the local culture and history in the Zhoushan islands and exhibit biologically interesting features, including a dark black coat colour and horns with various morphologies. In this study, we analysed the genetic relationship between Zhoushan cattle and other cattle and showed the genetic closeness of Zhoushan cattle to *B. indicus* bred in the nearby mainland regions. We also identified MC1R as a candidate gene responsible for the dark black coat colour of Zhoushan cattle. All these strategies and results provide us with valuable resources for developing strategies to conserve this unique cattle breed. Similarly, there are many outstanding local cattle breeds in China, and attention should be paid to their preservation. Conservation genomics is an effective strategy for preserving them.

In summary, we performed whole-genome sequencing of seven individuals of Zhoushan cattle and nine Wenling cattle individuals. Combined with the publicly-available genomic data of other cattle breeds, we identified that Zhoushan cattle is genetically similar to *B. indicus* in south-eastern China. Among them, Zhoushan cattle are most relative to Wenling cattle, suggesting that Zhoushan cattle's ancestor was *B. indicus* cattle bred in the mainland region close to the Zhoushan islands and introduced into the Zhoushan islands from there. Moreover, we identified MC1R as a candidate gene responsible for the dark black coat colour of Zhoushan cattle. Our results provide valuable resources for inferring the origin of Zhoushan cattle, characterising the uniqueness of Zhoushan cattle, and developing better breeding programs to preserve this unique and culturally important cattle breed.

## Methods

### Sample collection and whole-genome sequencing

We sampled a total of 16 cattle, including seven Zhoushan cattle and nine Wenling cattle. DNA was extracted from the ear tissues of each individual. DNA was extracted from the tissue using CTAB. Then, DNA quality and quantity were determined with an ND-2000 (NanoDrop Technologies). Only high-quality DNA samples (OD260/280 = 1.8–2.0, OD260/230 ≥ 2.0) were used to construct sequencing libraries. A DNA-seq library was prepared following the TruSeqTM Nano DNA Library Prep Kit from Illumina (San Diego, CA) using 1 μg of DNA that was fragmented by fragmentation buffer. Fragmented DNA was subjected to end-repair, phosphorylation, and 'A' base addition according to Illumina's library construction protocol. After quantification by TBS380, the paired-end DNA-seq library was sequenced with the Illumina HiSeq Xten/NovaSeq 6000 (2 × 150 bp read length).

### Read trimming and mapping to the reference genome

We trimmed the raw sequencing reads using Trimmomatic v0.38 (LEADING:30 TRAILING:30 SLIDINGWINDOW:4:25 MINLEN:50)^27^. We downloaded the UOA_Brahman_1 assembly of the cattle genome from Ensembl Release 101 (www.ensembl.org) and indexed it using BWA v0.7.16a-r1181^28^. The filtered reads were aligned to the UOA_Brahman_1 assembly by BWA-MEM with default parameters. We also downloaded the publicly-available whole-genome sequencing data of cattle breeds and analyzed them (Table S1). Unfortunately, body colour information of the five Angus individuals which we analyzed is not provided.

### Identification of genomic variants

We performed variant calling using samtools mpileup with default parameters^29^. We filtered out variants with a call rate < 80% and minor allele frequency < 5%. For variant annotations, the genome annotation of *B. indicus* annotation was obtained from the Ensembl Release 101 database. SNPs were annotated with SnpEff v4.2^30^. Finally, we retained 32,970,327 SNPs and 3,331,322 small indels as the initial dataset for the downstream analysis. The variant information was visualized with the Integrative Genomics Viewer^31^. In order to explore large structural variants in the MC1R region, we used samtools view to extract the reads mapped to the MC1R region (chr18:51,058,185–51,148,307 bp) and ran breakdancer-max v1.4.5. with parameter ‘-r 20’^21^.

### Population genomic analysis

The genomic variant data in VCF format was converted to PLINK binary format using PLINK v1.90b4.5^32^. PCA was performed with PLINK. The three-dimensional scatter plot was generated with the R rgl package. To build a NJ phylogenetic tree, we calculated pairwise genome-wide identical-by-state (IBS) distances based on the SNPs using PLINK. Based on the pairwise distance matrix (1-IBS), a NJ tree was constructed using MEGA7^33^. The admixture analysis was performed with ADMIXTURE v1.3.0 software (https://dalexander.github.io/admixture/index.html)^12^. Cross-validation (CV) errors were estimated for each K-value. The K-value with the lowest CV error was regarded as optimal for assessing the admixture level in each sample. The effective population size was calculated for each group using SNeP v1.1 with the maximum number of SNPs per chromosome at 10,000^34^. Average Fst values were calculated using a 40 kb window with 10 kb step using PLINK. Average Fst values greater than 0.6 were regarded as significant.

### Multiple sequence alignment of MC1R orthologs

The protein sequences of MC1R orthologs were downloaded from the Ensembl or NCBI Protein database. The identification numbers of all the sequences are listed in Table S3. These protein sequences were aligned by clustal omega with the default parameters^35^. The resulting multiple sequence alignments were verified and visualised by jalview^36^.

### Genome alignment

In order to compare the MC1R regions of the UOA_Angus_1 assembly (chr18:49,477,288–49,566,766 bp) and that of the UOA_Brahman_1 assembly (chr18:51,058,185–51,148,307 bp), we extracted the genome sequences of the MC1R regions from both assemblies. We aligned the sequence of the UOA_Angus_1 assembly to that of the UOA_Brahman_1 assembly by minimap2 and LASTZ^37^. The results were plotted by the plot function of R v3.5.2.

### Generation of the geographical maps

The geographical maps were generated by using R packages of maps v3.3.0 (https://cran.r-project.org/web/packages/maps) and mapdata v2.3.0 (https://cran.r-project.org/web/packages/mapdata).

### Ethics declarations

All procedures were performed in accordance with the guidelines of the Regulations for the Administration of Laboratory Animals (Decree No. 2 of the State Science and Technology Commission of the People's Republic of China, November 14, 1988) and were approved by the Animal Ethics Committee of Zhejiang Ocean University (Zhoushan, China). The authors declare that they have no known competing financial interests or personal relationships that could have appeared to influence the work reported in this paper.

## Supplementary Information

Supplementary Figure S1.

Supplementary Legends.

Supplementary Table S1.

Supplementary Table S2.

Supplementary Table S3.

## Data Availability

The raw reads were deposited in the DDBJ Sequence Read Archive (DRA) under accession number PRJDB10918.
