# Supplementary figures and images for: Whole-genome sequencing of endangered Zhoushan cattle suggests its origin and the association of MC1R with black coat colour
Source: Sci Rep. 2021 Aug 30;11:17359. doi: 10.1038/s41598-021-96896-2 (PMC8405626; doi:10.1038/s41598-021-96896-2)

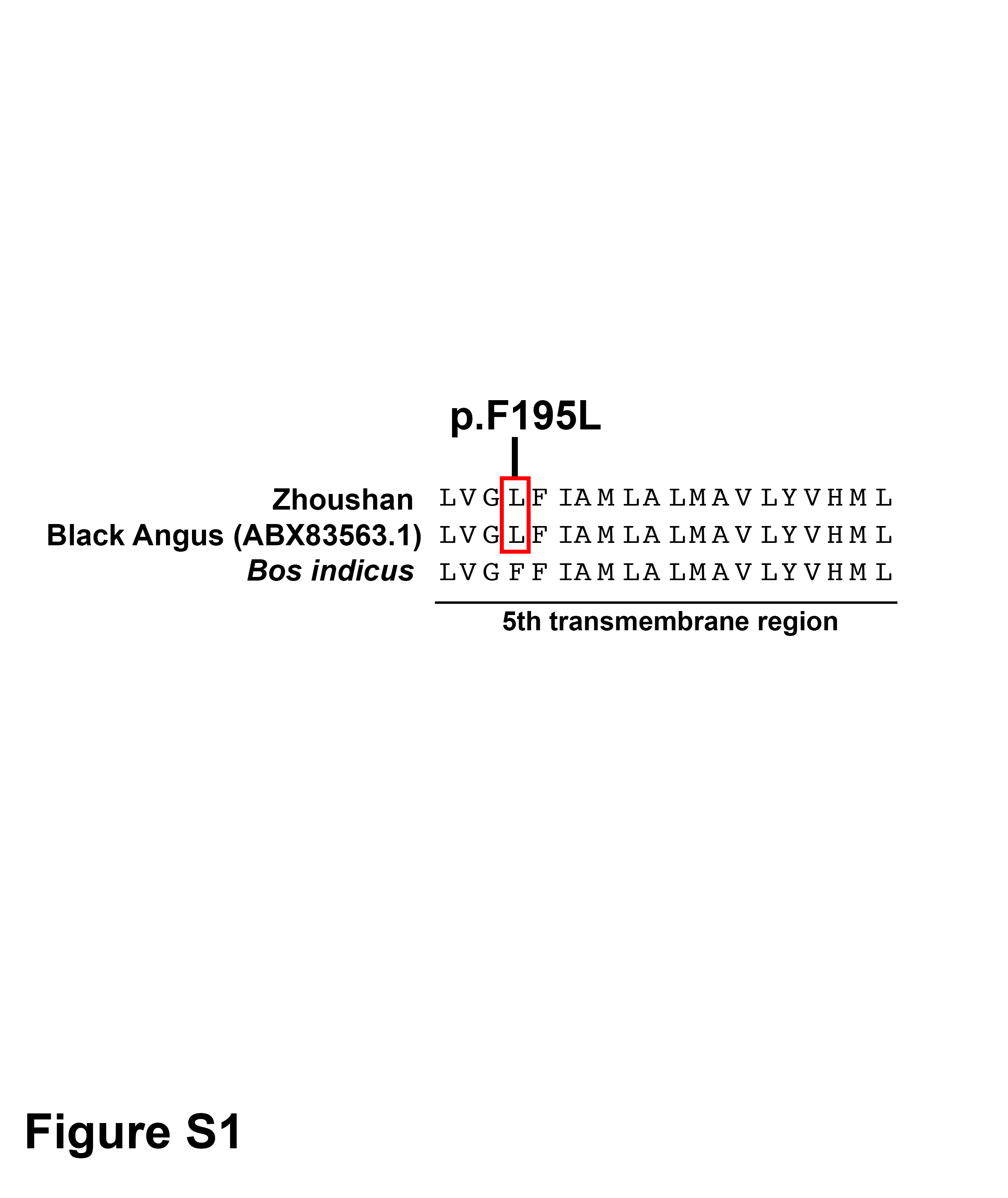

Supplement: Supplementary file 1 — Supplementary Figure S1. [file 41598_2021_96896_MOESM1_ESM.tif]
