# Supplementary material for: Whole-genome sequencing of endangered Zhoushan cattle suggests its origin and the association of MC1R with black coat colour
Source: Sci Rep. 2021 Aug 30;11:17359. doi: 10.1038/s41598-021-96896-2 (PMC8405626; doi:10.1038/s41598-021-96896-2)
Supplement: Supplementary file 2 — Supplementary Legends. [file 41598_2021_96896_MOESM2_ESM.docx]

**Supplementary information**

Figure S1. Sequence alignment of MC1R protein of Zhoushan cattle with that of Black Angus and *B. indicus*

Table S1. List of cattle individuals analysed in this study

Table S2. Mutations in the upstream or downstream regions of MC1R

Table S3. MC1R orthologs used in this study
